# Life-table studies revealed significant effects of deforestation on the development and survivorship of *Anopheles minimus* larvae

**DOI:** 10.1186/s13071-016-1611-5

**Published:** 2016-06-06

**Authors:** Xiaoming Wang, Guofa Zhou, Daibin Zhong, Xiaoling Wang, Ying Wang, Zhaoqing Yang, Liwang Cui, Guiyun Yan

**Affiliations:** School of Public Health and Tropical Medicine, Southern Medical University, Guangzhou, 510515 China; Program in Public Health, University of California at Irvine, Irvine, CA 92697 USA; National Institute of Parasitic Diseases, Chinese Center for Disease Control and Prevention, Shanghai, 200025 China; Institute of Tropical Medicine, Third Military Medical University, Chongqing, 400038 China; Department of Pathogen Biology, Kunming Medical University, Kunming, 650500 China; Department of Entomology, Pennsylvania State University, University Park, PA 16802 USA

**Keywords:** *Anopheles minimus*, Larvae, Deforestation, Life table, Mosquito, Malaria

## Abstract

**Background:**

Many developing countries are experiencing rapid ecological changes such as deforestation and shifting agricultural practices. These environmental changes may have an important consequence on malaria due to their impact on vector survival and reproduction. Despite intensive deforestation and malaria transmission in the China-Myanmar border area, the impact of deforestation on malaria vectors in the border area is unknown.

**Methods:**

We conducted life table studies on *Anopheles minimus* larvae to determine the pupation rate and development time in microcosms under deforested, banana plantation, and forested environments.

**Results:**

The pupation rate of *An. minimus* was 3.8 % in the forested environment. It was significantly increased to 12.5 % in banana plantations and to 52.5 % in the deforested area. Deforestation reduced larval-to-pupal development time by 1.9–3.3 days. Food supplementation to aquatic habitats in forested environments and banana plantations significantly increased larval survival rate to a similar level as in the deforested environment.

**Conclusion:**

Deforestation enhanced the survival and development of *An. minimus* larvae, a major malaria vector in the China-Myanmar border area. Experimental determination of the life table parameters on mosquito larvae under a variety of environmental conditions is valuable to model malaria transmission dynamics and impact by climate and environmental changes.

**Electronic supplementary material:**

The online version of this article (doi:10.1186/s13071-016-1611-5) contains supplementary material, which is available to authorized users.

## Background

Many developing countries are experiencing rapid ecological changes such as deforestation and shifting agricultural practices [1–4]. These ecological changes may have an important consequence on vector-borne disease transmission due to their impact on vector survival and reproduction [5–8]. In southeast Asia, including China and Myanmar, deforestation and cultivation of cash crops represent the most important environmental changes in rural areas [9, 10]. Many forests were depleted through illegal logging, agricultural clearing, and land development for housing and hydroelectric projects. Deforestation has led to major changes in the environment and subsequently may affect the ecology of malaria vectors. Deforestation may provide more favorable conditions for the larval development of anopheline species. For example, enhanced larval survivorship and faster larval-to-pupal development were found in *Anopheles gambiae* and *An. arabiensis* larvae in habitats from open-canopy areas in comparison to shaded habitats [11, 12]. It is possible that deforestation may be detrimental to the survival of other mosquito species. For example, *An. dirus* larvae breed in well shaded, temporary ground pools, or slow-moving water [13, 14]. These breeding habitats normally occur in the deep forest, making *An. dirus* a common vector at the forest edges [15]. Consequently, environmental disturbance such as deforestation can have positive or negative impacts on malaria vector populations, depending on the ecology of the vector species.

The malaria elimination goal set by China, Thailand and other countries in the Greater Mekong subregion faces a number of challenges [16, 17]. These challenges include parasite re-introduction by migratory human populations from the endemic neighboring country Myanmar. For example, among malaria patients over 15 years old in Yunnan, imported cases constituted 22 % of total cases in men and 13 % in women, and Myanmar was the predominant source of infection [18]. The second is intense malaria transmission in the remote border areas where impoverished minority ethnic groups are concentrated and resources for malaria control are very limited [19]. In the border area between China and Myanmar, illegal logging of the tropical rainforest and forest clearing for cash crops have been particularly severe [20, 21]. There is no information on how these environmental changes alter the ecology of malaria vectors in the border area. However, this knowledge is critical to malaria control in the border area where malaria burden is the highest and residents are most prone to malaria epidemics.

The objective of this study is to determine the impact of deforestation and agricultural land development on the larval ecology of *An. minimus,* a key malaria vector species in China-Myanmar and Thai-Myanmar border areas. Knowledge of the response to anopheline vectors to environmental changes will provide a better understanding of malaria transmission dynamics, which is crucial for implementing effective malaria control strategies.

## Methods

### Study sites

**Fig. 1:**
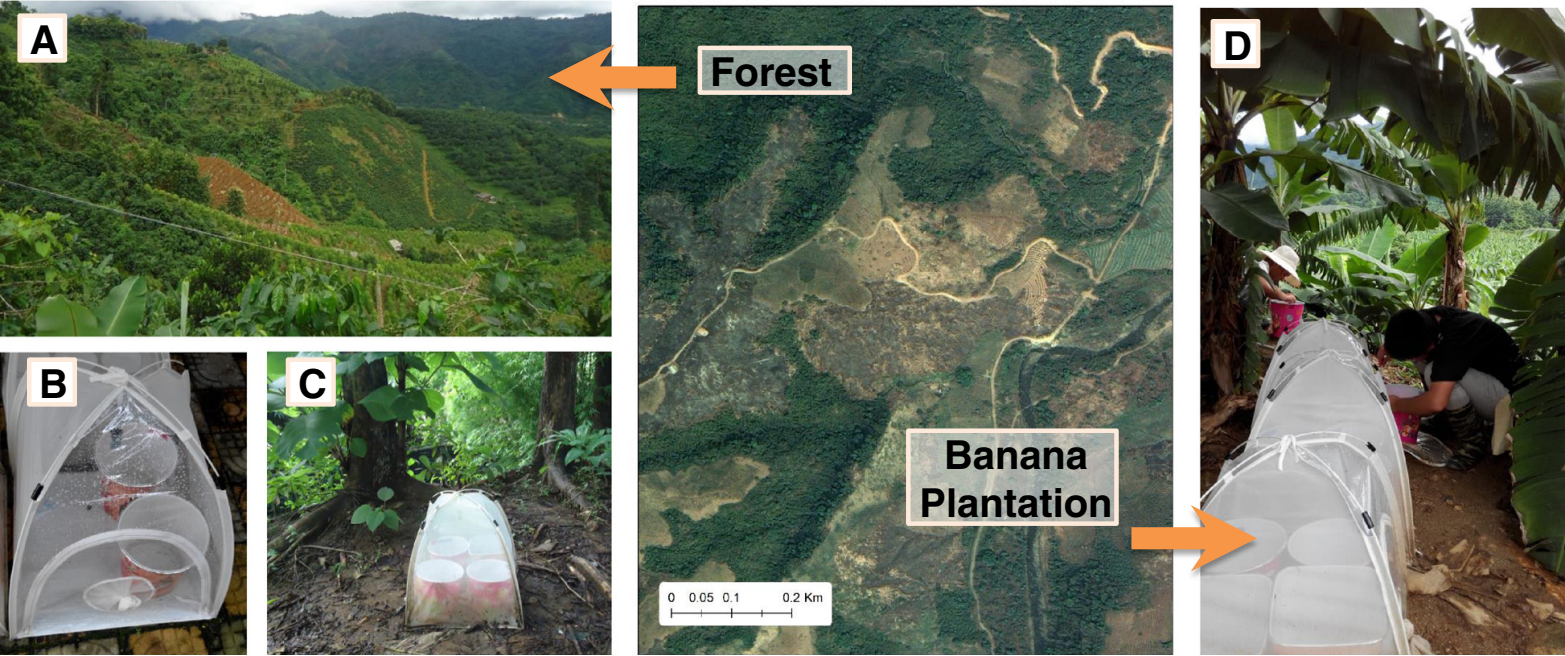
Pictures of field settings. Deforestation and plantation of banana, rubber and other plants in the study site (**a**), and microcosms inside a InsectDorm placed under a deforested area (**b**), forested area (**c**), and banana field (**d**)

The study was conducted in Nabang (24°45′ E, 97°32′ N), Yingjiang County, Yunnan Province, China on China-Myanmar border area. Deforestation represents the most important environmental change in the area (Fig. 1). The deforested areas were often converted to maize, banana, or rubber plantations. In this study, we selected three land use and land cover types: forested areas, banana fields (cultivated after deforestation), and newly deforested areas. A forested area is defined as an area with tree canopy over 60 %; and a deforested area is an area that has less than 10 % canopy coverage but where a plantation has not yet been started [5]. The forested and deforested areas were within 1 km distance at the same elevation (250 m above sea level) (Fig. 1).

### Experiments

Blood-engorged *Anopheles* adult mosquitoes were collected from the deforested area and separated to species by morphology. F1 eggs were collected and allowed to hatch in the insectary. The female parents were collected for molecular identification using the multiplex polymerase chain reaction (PCR) method [22, 23]. Because *An. minimus* was the predominant vector species in the field collection, it was the focus of the present study. We constructed microcosms using plastic bins of 24 cm in diameter and 27 cm deep (Fig. 1). Two liters of rainwater were added to the plastic bin and allowed to acclimate for 1 day. To prevent other insects from invading the microcosms or other mosquitoes from laying eggs, the microcosms were placed in an insect-proof 61 × 61 × 61 cm^3^ BugDorm tent (BioQuip, Rancho Dominguez, CA, USA) (Fig. 1). All sides of the BugDorm tent were made of clear polyester netting materials, so that sunlight was not blocked. Microcosms mimicked natural habitats, and were homogeneous. The homogeneous feature of microcosms had the advantage over the natural habitats which were highly variable in habitat size, larval food conditions (e.g. organic matters), vegetation coverage and predators.

Fifty newly hatched *An. minimus* larvae were introduced to each microcosm. Each day the number of surviving larvae was counted and their stage of development recorded. Pupae were counted and removed daily. Water levels in the microcosm were checked daily, and were maintained by adding rainwater if needed. Water temperature was measured using the HOBO data loggers placed in the microcosm, 1 cm below the water surface. There were 8 microcosms in each of the three land use and land cover types (deforested, banana field and forest area). Microcosms in the forested environment may not receive sufficient sunlight for microbial growth and thus mosquito larvae failed to develop due to a lack of necessary nutrients. To test this hypothesis, 12 microcosms (4 replicates per land use type) were supplemented with Tetramin fish food daily, and daily larval mosquito survivorship was measured.

### Data analysis

The pupation rate of *An. minimus* larvae was calculated as the proportion of 1^st^ instar larvae that developed into pupae. Mean larval-to-pupal development time was calculated. The *t-*test and analysis of variance (ANOVA) were used to determine the effect of land use and land cover types and larval food supplementation on pupation rate and larval development time wherever appropriate. Kaplan-Meier survival analysis was performed to determine the effects on larval survivorship. The log-rank test was used to test the statistical significance. Stage-specific larval development time and mortality rate were calculated. All data analyses were performed using STATISTICA 10.0 (StatSoft, Tulsa, USA).

## Results

### Field-collected mosquito species

A total of 218 blood-engorged *Anopheles* adult mosquitoes were collected from indoors and outdoors (pig and cow shelter) using aspirators. Among these, 94 % were *An*. *minimus* and the remaining mosquitoes were *An. maculatus.* The PCR method confirmed morphological identification.

### Effects of land use and land cover on *An. minimus* larvae

**Fig. 2:**
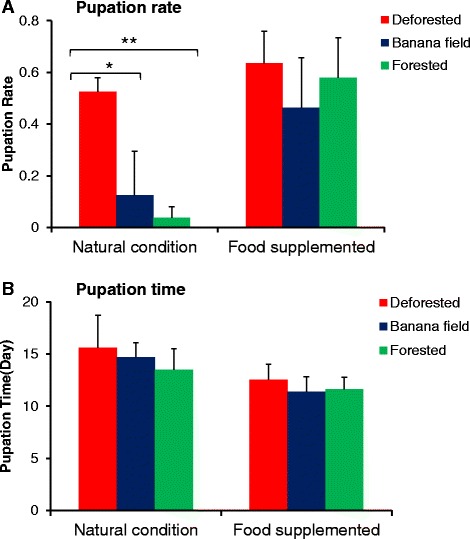
Pupation rates (**a**) and larval-to-pupal development time (**b**) of *Anopheles minimus* larvae in three land use and land cover conditions. Two experimental conditions were used: natural condition (*left*) and larval food supplementation (*right*)

**Fig. 3:**
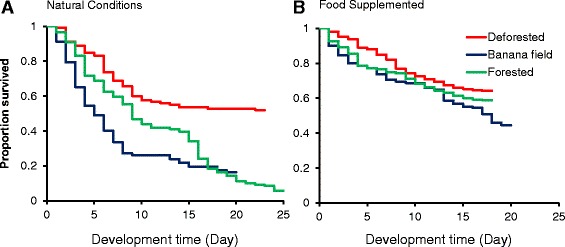
Kaplan-Meier survival curve of *Anopheles minimus* larvae in three land use and land cover conditions. **a** natural conditions; and **b** larval food supplemented

Under natural conditions, 52.5 % of the 1^st^ instar larvae developed to pupae in the deforested area, whereas pupation rate was significantly reduced in the banana field (12.5 %) (*t*_(1)_ *=* 4.48*, P =* 0.01) and the forested area (3.8 %) (*t*_(1)_ = 14.19, *P <* 0.0001) (Fig. 2a) (Additional file 1). Pupation rate was not significantly different between the banana field and the forest (*t*_(1)_ = 0.99*, P* = 0.39). The stage-specific mortality rate was high in the 1^st^ and 2^nd^ instar larvae (Table 1). This was consistent with the Kaplan-Meier analysis of larval survivorship which showed higher mortality in young larvae than older larvae (Fig. 3). In the forested environment, there was a high mortality in the 4^th^-instar larvae: 34 % of the 4^th^ instar larvae failed to develop into pupae (Table 1). Among those that successfully developed into pupae, the larval-to-pupal development time was about 13.5–15.6 days, and it was not significantly different among the three land cover types (ANOVA, *F*_(2, 7)_ = 0.58, *P* = 0.59) (Fig. 2b).

**Table 1:** Developmental stage-specific survivorship of immature *An. minimus* under three land use and land cover environments under natural condition and larval food supplementation

		Natural conditions			Food supplemented		
Land use	Stage	Development time (day)	Cumulative survival rate	Stage mortality rate	Development time (day)	Cumulative survival rate	Stage mortality rate
Deforested	1st instar	2.81	0.91	0.08	3.03	0.98	0.09
	2nd instar	3.66	0.83	0.18	3.34	0.89	0.07
	3rd instar	5.15	0.65	0.10	3.97	0.82	0.11
	4th instar	4.01	0.55	0.03	2.17	0.71	0.07
Banana field	1st instar	3.1	0.79	0.25	2.98	0.90	0.11
	2nd instar	5.12	0.54	0.28	3.63	0.79	0.08
	3rd instar	3.17	0.26	0.00	2.85	0.71	0.02
	4th instar	3.28	0.26	0.10	1.93	0.69	0.24
Forested	1st instar	3.44	0.91	0.19	2.72	0.93	0.14
	2nd instar	3.31	0.72	0.16	3.13	0.79	0.04
	3rd instar	6.36	0.56	0.16	3.51	0.75	0.07
	4th instar	0.31	0.40	0.34	2.24	0.68	0.09

### Effects of food supplementation

With food supplementation, pupation rate increased significantly for all three land use and land cover types (Fig. 2a). A particularly large change was found in banana fields and forested environments where a 3.7 fold (*t*_(1)_ = 2.92*, P* = 0.02) and 15.4 fold (*t*_(1)_ = 9.26*, P* < 0.0001) increase was found in comparison to the natural conditions. The pupation rate did not vary significantly among the three land use and land cover types when larval food was added to the microcosms, suggesting that sunlight contributed significantly to the process of larval food production in the habitats. Stage-specific mortality analysis found a dramatic reduction in the mortality rate for all 4 larval instars (Table 1). The average larval-to-pupal development pupation time did not vary significantly among the three land cover types. Comparing the treatment without food supplementation, food supplementation reduced larval development time by 3.1 days (*t*_(1)_ = 1.66*, P* = 0.17) in the deforested area, by 3.3 days in the banana field (*t*_(1)_ = 2.74*, P* = 0.05) and by 1.9 days (*t*_(1)_ = 1.25, *P* = 0.34) in the forested environment (Fig. 2b). Kaplan-Meier survivor analysis found similar mortality rate across time for the three land use and land cover types (Fig. 3).

### Effects of land use and land cover on habitat temperature

**Fig. 4:**
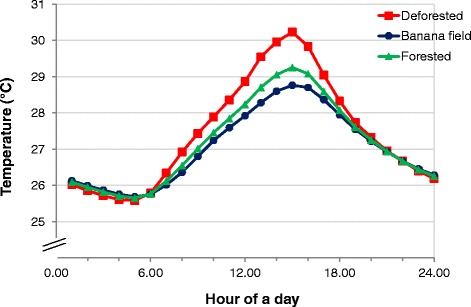
Mean hourly temperature 24-hr daily cycle in microcosms in three land use and land cover conditions

The average temperature of the microcosms in the deforested environment was significantly higher than the banana fields (*t*_(1)_ = 1.95*, P* = 0.05). A larger fluctuation in hourly temperature was observed in the deforested environment (Fig. 4). Overall, the minimum and maximum temperature ranged from 25.5 to 30.2 °C, within the optimal temperature for larval mosquito development.

## Discussion

The present study found that in comparison to larval habitats in the forested area, the pupation rate of *An. minimus* was about 4-fold higher in banana plantation fields and 13-fold higher in deforested area, and the larval-to-pupal development time was shortened by 1.9–3.3 days. The microcosms were covered by insect-proof materials and no predator had been observed in the microcosms. Because the experiments used microcosms made of the same rain water and same substrate, and the only difference was the environment in which the microcosms were placed (forested, banana field and deforested); the difference in pupation rate and development time of mosquito larvae among the three environments was entirely due to land use and land cover differences. This finding was consistent with the study in the Peruvian Amazon where *An. darlingi* larvae were more commonly found in open sunlit aquatic habitats [6]. Tuno et al. demonstrated that the survivorship of *An. gambiae* larvae was reduced from 55 to 57 % in habitats fully exposed to sunlight to 1–2 % in habitats with full forest canopy coverage (forest habitats) in western Kenya highlands [24]. Whether the observation that deforestation facilitates the survival of the immature stage of anopheline mosquitoes is a common phenomenon across various malaria vector species and across multiple continents warrants further investigation.

The mechanisms for reduced larval survivorship in microcosms placed in the forested environment and banana plantation with high canopy coverage are unknown. Land use and land cover may modify the temperature and food source availability for mosquito larvae. We observed an average of 0.4 °C lower temperature in microcosms in the forested environment than deforested environment although the minimum and maximum temperature in forested and sunlit deforested microcosms ranged between 25.5–30.2 °C, within the optimal temperature for mosquito development. Lower pupation rate in habitats from the forest environment could lead to large early-instar larval mortality, subsequently leading to lower pupation rate as observed in *Culex quinquefasciatus* [25]. The larval density used in the present study was comparable to the density in natural habitats. Field sampling in over 100 habitats of various habitat types found that the mean density of 3^rd^ and 4^th^ instar *Anopheles* larvae ranged from 1.5–2 per dipper (300 ml volume), or 10–13 larvae per 2 l water (unpublished data). In the present experiments we observed ~20–40 % of the larvae developed into 3^rd^ or 4^th^ instar (or 8–20 larvae in 2 l microcosms). Further, the same larval density used in all experiments enabled us to compare the environmental impact on larval development and survivorship. Another hypothesis is the lack of food for mosquito larvae in the shaded microcosms. This hypothesis was supported by food supplementation to shaded microcosms and resulting recuperation of mosquito pupation rate to a level comparable to the deforested environment. Additionally, faster larval-to-pupal development was found in all three land use and land cover types. The potential food source of anopheline larvae may include bacteria, fungi, diatom, detritus, organic matter, and others. Using stable isotopes of carbon and nitrogen, Gilbreath et al. demonstrated inter-specific resource partitioning between *Culex quinquefasciatus* and *An. gambiae* larvae in natural habitats in western Kenya [26], but resource utilization of bacteria, algae, organic matter or others by *Anopheles* larvae and their nutritional contribution to mosquito larval biomass are virtually unknown. The abundance and structure of microbes such as algae and photosynthetic cyanobacteria in aquatic habitats may have changed in response to shading [27]. Information on the contribution of specific microbes to larval mosquito development and survival will significantly enhance our understanding of larval ecology.

Our finding on the impact of environmental changes on larval development of malaria vectors has important implications on understanding malaria transmission dynamics in the China-Myanmar border area. First, *An. minimus* larvae can successfully develop into pupae and adults and complete its life cycle in the forest despite of much reduced pupation rate. If aquatic habitats are abundant in the forest, a considerable number of adult mosquitoes may be produced. Because *An. minimus* is highly anthropophilic and highly susceptible to malaria parasites [28, 29], this presents a significant risk to forest workers and hunters. Secondly, significantly enhanced larval survivorship and pupation rate in *An. minimus* mosquitoes in the deforested and cultivated areas render the local residents highly prone to malaria outbreaks because more human-mosquito contact is expected in such environments. Therefore, close malaria surveillance for local residents living near deforested and cultivated areas is needed to prevent outbreaks. Thirdly, climate warming is expected to affect the ecology of *Anopheles* mosquitoes and their malaria transmission potential. It is imperative to use various tools, including mathematical modeling to examine the impact of climate and environmental changes on vector and malaria transmission dynamics [30]. Larval survivorship and development time are important parameters for modeling transmission dynamics. The present study provided valuable information on these important parameters.

This study has several limitations. First, we examined the response of one of the most abundant malaria vector species to environmental changes. There are multiple malaria vectors in the area, including *An. maculatus*, *An. culicifacies*, *An. dirus* and others [23]. It is possible that other vector species may respond to environmental changes differently. It would be particularly interesting to study the mechanisms and larval ecology of *An. dirus* that was found exhibiting reduced abundance in deforested areas. Second, we did not examine the effect of deforestation on the survival and reproduction of adult mosquitoes. Enhanced survivorship and blood feeding frequency in adult mosquitoes by deforestation through the effects on the microclimatic condition of the mosquitoes were found in other *Anopheles* species [31], and it is possible that such effects are also manifested in *An. minimus* adults. Third, the study was conducted in microcosms. The findings from the present study need validating in natural habitats.

## Conclusions

The present study shows that deforestation significantly enhanced the pupation rate and shortened the larval-to-pupal development time of *An. minimus* larvae, a major malaria vector in southeast Asia. The results of the present study on the life table parameters of mosquitoes under a variety of environmental conditions is valuable to model malaria transmission dynamics and the impact by climate and environmental changes in southeast Asia.

## Abbreviations

ANOVA, analysis of variance; PCR, polymerase chain reaction.

## Additional file

Additional file 1: Raw data on the survivorship of *Anopheles minimus* larvae in three land use and land cover conditions under natural conditions and with larval food supplemented. (DOCX 13 kb)

## References

[1] Abdullah SA, Hezri AA (2008). From forest landscape to agricultural landscape in the developing tropical country of Malaysia: pattern, process, and their significance on policy. Environ Manag.

[2] Chua KB, Chua BH, Wang CW (2002). Anthropogenic deforestation, El Nino and the emergence of Nipah virus in Malaysia. Malays J Pathol.

[3] Garedew E, Sandewall M, Soderberg U, Campbell BM (2009). Land-use and land-cover dynamics in the central rift valley of Ethiopia. Environ Manag.

[4] Rudel TK (2013). The national determinants of deforestation in sub-Saharan Africa. Philos Trans R Soc Lond B Biol Sci.

[5] Afrane YA, Little TJ, Lawson BW, Githeko AK, Yan G (2008). Deforestation and vectorial capacity of Anopheles gambiae Giles mosquitoes in malaria transmission, Kenya. Emerg Infect Dis.

[6] Vittor AY, Pan W, Gilman RH, Tielsch J, Glass G, Shields T (2009). Linking deforestation to malaria in the Amazon: characterization of the breeding habitat of the principal malaria vector, Anopheles darlingi. Am J Trop Med Hyg.

[7] Walsh JF, Molyneux DH, Birley MH (1993). Deforestation: effects on vector-borne disease. Parasitology.

[8] Chaves LF, Koenraadt CJ (2010). Climate change and highland malaria: fresh air for a hot debate. Q Rev Biol.

[9] Hillman AL, Yu JQ, Abbott MB, Cooke CA, Bain DJ, Steinman BA (2014). Rapid environmental change during dynastic transitions in Yunnan Province, China. Quaternary Sci Rev.

[10] Webb EL, Jachowski NRA, Phelps J, Friess DA, Than MM, Ziegler AD (2014). Deforestation in the Ayeyarwady Delta and the conservation implications of an internationally-engaged Myanmar. Global Environ Chang.

[11] Munga S, Minakawa N, Zhou G, Mushinzimana E, Barrack OO, Githeko AK (2006). Association between land cover and habitat productivity of malaria vectors in western Kenyan highlands. Am J Trop Med Hyg.

[12] Afrane YA, Zhou G, Lawson BW, Githeko AK, Yan G (2007). Life-table analysis of *Anopheles arabiensis* in western Kenya highlands: effects of land covers on larval and adult survivorship. Am J Trop Med Hyg.

[13] Rattanarithikul R, Green CA, Panyim S, Noigamol C, Chanaimongkol S, Mahapibul P (1995). Larval habitats of malaria vectors and other *Anopheles* mosquitoes around a transmission focus in northwestern Thailand. J Am Mosq Control Assoc.

[14] Rosenberg R, Maheswary NP (1982). Forest malaria in Bangladesh. II. Transmission by *Anopheles dirus*. Am J Trop Hyg.

[15] Obsomer V, Defourny P, Coosemans M (2007). The Anopheles dirus complex: spatial distribution and environmental drivers. Malar J.

[16] Hsiang MS, Gosling RD (2015). Striding toward malaria elimination in China. Am J Trop Med Hyg.

[17] Maude RJ, Lubell Y, Socheat D, Yeung S, Saralamba S, Pongtavornpinyo W (2010). The role of mathematical modelling in guiding the science and economics of malaria elimination. Int Health.

[18] Lin H, Lu L, Tian L, Zhou S, Wu H, Bi Y (2009). Spatial and temporal distribution of falciparum malaria in China. Malar J.

[19] Cui L, Yan G, Sattabongkot J, Cao Y, Chen B, Chen X (2012). Malaria in the Greater Mekong Subregion: heterogeneity and complexity. Acta Trop.

[20] Achard F, Eva HD, Stibig HJ, Mayaux P, Gallego J, Richards T (2002). Determination of deforestation rates of the world's humid tropical forests. Science.

[21] Gibbs HK, Ruesch AS, Achard F, Clayton MK, Holmgren P, Ramankutty N (2010). Tropical forests were the primary sources of new agricultural land in the 1980s and 1990s. Proc Natl Acad Sci U S A.

[22] Wang Y, Zhong D, Cui L, Lee MC, Yang Z, Yan G (2015). Population dynamics and community structure of *Anopheles* mosquitoes along the China-Myanmar border. Parasit Vectors.

[23] Yu G, Yan G, Zhang N, Zhong D, Wang Y, He Z (2013). The *Anopheles* community and the role of *Anopheles minimus* on malaria transmission on the China-Myanmar border.. Parasit Vectors.

[24] Tuno N, Okeka W, Minakawa N, Takagi M, Yan G (2005). Survivorship of *Anopheles gambiae* sensu stricto (Diptera: Culicidae) larvae in western Kenya highland forest. J Med Entomol.

[25] Chaves LF, Keogh CL, Vazquez-Prokopec GM, Kitron UD (2009). Combined sewage overflow enhances oviposition of *Culex quinquefasciatus* (Diptera: Culicidae) in urban areas. J Med Entomol.

[26] Gilbreath TM, Kweka EJ, Afrane YA, Githeko AK, Yan G (2013). Evaluating larval mosquito resource partitioning in western Kenya using stable isotopes of carbon and nitrogen. Parasit Vectors.

[27] Wang Y, Gilbreath TM, Kukutla P, Yan G, Xu J (2011). Dynamic gut microbiome across life history of the malaria mosquito *Anopheles gambiae* in Kenya. PLoS One.

[28] Polsomboon S, Poolprasert P, Suwonkerd W, Bangs MJ, Tanasinchayakul S, Akratanakul P (2008). Biting patterns of *Anopheles minimus* complex (Diptera: Culicidae) in experimental huts treated with DDT and deltamethrin. J Vector Ecol.

[29] Tisgratog R, Tananchai C, Juntarajumnong W, Tuntakom S, Bangs MJ, Corbel V (2012). Host feeding patterns and preference of *Anopheles minimus* (Diptera: Culicidae) in a malaria endemic area of western Thailand: baseline site description. Parasit Vectors.

[30] Reiner RC, Perkins TA, Barker CM, Niu T, Chaves LF, Ellis AM (2013). A systematic review of mathematical models of mosquito-borne pathogen transmission: 1970-2010. J R Soc Interface.

[31] Afrane YA, Lawson BW, Githeko AK, Yan G (2005). Effects of microclimatic changes caused by land use and land cover on duration of gonotrophic cycles of *Anopheles gambiae* (Diptera: Culicidae) in western Kenya highlands. J Med Entomol.

