# Supplementary material for: Life-table studies revealed significant effects of deforestation on the development and survivorship of Anopheles minimus larvae
Source: Parasit Vectors. 2016 Jun 6;9:323. doi: 10.1186/s13071-016-1611-5 (PMC4895827; doi:10.1186/s13071-016-1611-5)
Supplement: Additional file 1: — Raw data on the survivorship of Anopheles minimus larvae in three land use and land cover conditions under natural conditions and with larval food supplemented. (DOCX 13 kb) [file 13071_2016_1611_MOESM1_ESM.docx]

**Table S1:** Survivorship of *Anopheles minimus* larvae in three land use and land cover conditions under natural conditions and with larval food supplemented.

|  | Natural Conditions | | |  | Larval Food Supplemented | | |
| --- | --- | --- | --- | --- | --- | --- | --- |
| Day | Deforested | Banana field | Forested |  | Deforested | Banana field | Forested |
| 0 | 1.000 | 1.000 | 1.000 |  | 1.000 | 1.000 | 1.000 |
| 1 | 0.992 | 0.913 | 0.965 |  | 0.979 | 0.900 | 0.926 |
| 2 | 0.912 | 0.793 | 0.908 |  | 0.952 | 0.845 | 0.891 |
| 3 | 0.888 | 0.652 | 0.830 |  | 0.938 | 0.800 | 0.855 |
| 4 | 0.848 | 0.543 | 0.716 |  | 0.890 | 0.786 | 0.785 |
| 5 | 0.832 | 0.489 | 0.688 |  | 0.880 | 0.773 | 0.772 |
| 6 | 0.736 | 0.402 | 0.624 |  | 0.849 | 0.736 | 0.765 |
| 7 | 0.688 | 0.337 | 0.596 |  | 0.821 | 0.705 | 0.749 |
| 8 | 0.656 | 0.272 | 0.553 |  | 0.770 | 0.695 | 0.743 |
| 9 | 0.600 | 0.261 | 0.468 |  | 0.742 | 0.686 | 0.711 |
| 10 | 0.576 | 0.261 | 0.440 |  | 0.725 | 0.686 | 0.682 |
| 11 | 0.568 | 0.261 | 0.418 |  | 0.708 | 0.659 | 0.662 |
| 12 | 0.560 | 0.261 | 0.418 |  | 0.694 | 0.650 | 0.643 |
| 13 | 0.552 | 0.239 | 0.411 |  | 0.674 | 0.586 | 0.630 |
| 14 | 0.536 | 0.217 | 0.397 |  | 0.660 | 0.568 | 0.614 |
| 15 | 0.536 | 0.196 | 0.340 |  | 0.653 | 0.550 | 0.601 |
| 16 | 0.536 | 0.196 | 0.241 |  | 0.646 | 0.545 | 0.592 |
| 17 | 0.528 | 0.196 | 0.184 |  | 0.643 | 0.509 | 0.588 |
| 18 | 0.528 | 0.174 | 0.163 |  |  | 0.459 |  |
| 19 | 0.528 | 0.163 | 0.142 |  |  | 0.445 |  |
| 20 | 0.528 |  | 0.113 |  |  |  |  |
| 21 | 0.528 |  | 0.099 |  |  |  |  |
| 22 | 0.520 |  | 0.092 |  |  |  |  |
| 23 |  |  | 0.085 |  |  |  |  |
| 24 |  |  | 0.057 |  |  |  |  |
